# Improving quality in population surveys of headache prevalence, burden and cost: key methodological considerations

**DOI:** 10.1186/1129-2377-14-87

**Published:** 2013-10-25

**Authors:** Timothy J Steiner, Lars Jacob Stovner, Mohammed Al Jumah, Gretchen L Birbeck, Gopalakrishna Gururaj, Rigmor Jensen, Zaza Katsarava, Luiz Paulo Queiroz, Ann I Scher, Redda Tekle-Haimanot, Shuu-Jiun Wang, Paolo Martelletti, Tarun Dua, Somnath Chatterji

**Affiliations:** 1 Norwegian National Headache Centre, Norwegian University of Science and Technology, and St Olavs University Hospital, Trondheim, Norway; 2 Department of Neuroscience, Imperial College London, London, UK; 3 King Saud Bin Abdul-Aziz University for Health Sciences, Riyadh, Saudi Arabia; 4 Department of Neurology, University of Rochester, Rochester, NY, USA; 5 Chikankata Hospital, Mazabuka, Zambia; 6 Department of Epidemiology, Centre for Public Health, National Institute of Mental Health and Neuro Sciences, Bangalore, India; 7 Danish Headache Centre, Glostrup Hospital, University of Copenhagen, Copenhagen, Denmark; 8 Evangelical Hospital, Unna, Germany; 9 Department of Neurology, University of Duisburg-Essen, Essen, Germany; 10 Department of Neurology, University Hospital, Federal University of Santa Catarina, Florianopolis, Brazil; 11 Uniformed Services University, Bethesda, MD, USA; 12 School of Medicine, Department of Neurology, Addis Ababa University, Addis Ababa, Ethiopia; 13 The Neurological Institute, Taipei Veterans General Hospital, Taipei, Taiwan; 14 Department of Neurology, Brain Research Center and Institute of Brain Science, National Yang-Ming University of School of Medicine, Taipei, Taiwan; 15 Department of Clinical and Molecular Medicine, Sapienza University of Rome, Rome, Italy; 16 Department of Mental Health and Substance Abuse, World Health Organization, Geneva, Switzerland; 17 Department of Health Statistics and Informatics, World Health Organization, Geneva, Switzerland

**Keywords:** Epidemiology, Burden of headache, Methodology, Global Campaign against Headache

## Abstract

Population-based studies of headache disorders are important. They inform needs assessment and underpin service policy for a set of disorders that are a public-health priority. On the one hand, our knowledge of the global burden of headache is incomplete, with major geographical gaps; on the other, methodological differences and variable quality are notable among published studies of headache prevalence, burden and cost.

The purpose here was to start the process of developing standardized and better methodology in these studies. An expert consensus group was assembled to identify the key methodological issues, and areas where studies might fail. Members had competence and practical experience in headache epidemiology or epidemiology in general, and were drawn from all WHO world regions. We reviewed the relevant literature, and supplemented the knowledge gathered from this exercise with experience gained from recent Global Campaign population-based studies, not all yet published. We extracted methodological themes and identified issues within them that were of key importance.

We found wide variations in methodology. The themes within which methodological shortcomings had adverse impact on quality were the following: study design; selection and/or definition of population of interest; sampling and bias avoidance; sample size estimation; access to selected subjects (managing and reporting non-participation); case definition (including diagnosis and timeframe); case ascertainment (including diagnostic validation of questionnaires); burden estimation; reporting (methods and results). These are discussed.

## Introduction

Epidemiological studies of headache are important. They enhance our understanding of its origins, patterns, aetiology and risk factors, so improving opportunities for treatment and prevention of headache. They inform needs assessment, underpin service policy and push for acceptance of headache disorders as a public-health priority. They gain in importance because headache disorders are themselves important, a fact greatly emphasized by the Global Burden of Disease Study 2010 (GBD2010) led by the Institute of Health Metrics and Evaluation, which placed headache disorders among the top ten causes of disability worldwide [1].

GBD2010 measured “burden” in terms of disability-adjusted life years (DALYs), a metric derived by summing years of life lost to premature mortality (YLLs) and years lost to disability (YLDs). Only the latter are relevant to headache disorders. These are undoubtedly useful concepts for making comparisons between diseases, informative especially for health policy formulation and health-care resource allocation, but they are narrow measures nonetheless. We use “burden of disease” to mean all the negative consequences of living with a disease, although in practice not all are measurable.

Our account of the global burden of headache is incomplete. The numerous published studies of headache prevalence, and the not so many of burden, are notably focused on the western world and on migraine [2]. The Global Campaign against Headache has been active in redressing this [3-6], but the evidence adduced for GBD2010 had major gaps, especially in African, South-East Asia and Eastern Mediterranean Regions. In addition, methodological differences and variable quality have both been major factors influencing findings among the studies reviewed by Stovner *et al. *[2]. While more population-based studies of headache disorders are certainly called for, the need for standardized – and better – methodology in such studies has long been evident [2,7].

The purpose of this document, a product of the Global Campaign against Headache [7-9], is to start the process of meeting this need. In highlighting the key methodological issues, it draws attention to areas where studies may fail, despite commitment to them of much money and time. It concentrates on issues that relate specifically or are of particular relevance to headache; discussion of general epidemiological principles is avoided. Even more, the focus is on headache disorders of public-health interest: migraine and tension-type headache (TTH). Medication-overuse headache (MOH) is also included because, on present understanding, it arises only as a complication of a pre-existing headache disorder, usually migraine. Unquestionably, MOH contributes to public ill-health [8,9].

### Procedure

The process was led by *Lifting The Burden* (LTB), a non-governmental organization conducting the Global Campaign against Headache in official relations with the World Health Organization (WHO) [10-13]. LTB assembled an expert consensus group (the co-authors), selecting members to bring experience and competence in headache epidemiology and/or epidemiology in general, to include WHO staff members with expertise in these fields, and, in pursuit of international and cross-cultural relevance, to have personal knowledge of all six WHO world regions.

We reviewed the relevant literature. Reviews of the world literature on headache epidemiology had been performed already, in earlier initiatives to document the prevalence and burden of headache [2,14-16] and the methodological issues arising from their measurement [7]. These reviews had been updated by LJS and TJS in the process of submitting evidence for GBD2010 [1]. We supplemented the knowledge gathered from these with more recent experience gained from LTB-supported population-based studies: four completed in Russia [3], China [4], India [5] and Pakistan [6], and three others still in progress and not yet published in Saudi Arabia, Zambia and Ethiopia. We extracted methodological themes and, within these, identified the areas in which methodological shortcomings with detrimental effect on quality were evident.

The expert group first acted as a sounding board in this process, conducted initially by email, and then convened as a consensus group at a meeting in Trondheim in August 2011. Through critical review, we applied a process of item reduction and distillation, retaining only those themes and issues arising from them that we considered of key importance. Final consensus was achieved through further email exchanges.

## Results

As anticipated, we found wide variations in reported methodology. The themes within which, in our view, methodological shortcomings had adverse impact on quality were the following:

• Study design;

• Selection and/or definition of population of interest;

• Sampling and bias avoidance;

• Sample size estimation;

• Access to selected subjects (managing and reporting non-participation);

• Case definition (including diagnosis and timeframe);

• Case ascertainment (including diagnostic validation of questionnaires);

• Burden estimation;

• Reporting (methods and results).

Our findings are summarized under these themes in Table 1, and are expanded below. Where we give examples, these are from studies that were generally of good quality but fell down in specific areas; we did not make a register of studies that were overall of poor quality.

**Table 1:** Methodological themes from the literature review, and shortcomings detrimental to quality

**Theme**	**Methodological issue**	**Potential impact on quality**
Study design	Inappropriately selected for overall purpose	Very high
	Unsuited to secondary purpose(s)	Low
Population of interest	Inappropriately selected for purpose	Very high
	Inadequately defined	High or very high
Sampling method (including means of access)	Systematically bias generating	Moderate to high
	Inadequately reported	High
Sample size	Inadequate for purpose	Low to high
Non-participation	Excessively reducing sample size	Low to high
	Bias generating	Moderate to high
	Inadequately reported	High or very high
Case definition	Not according to accepted diagnostic criteria	Moderate to high
	Inadequately reported	High or very high
	Applying inappropriate or undefined timeframe	High or very high
Case ascertainment	Not according to accepted diagnostic criteria	Moderate to high
	Employing unvalidated diagnostic methods	Moderate to high
	Inadequately reported	High
Burden estimation	Not relevant	Low to moderate
	Not comprehensive	Low
Reporting	Not adequately descriptive of methods	Low to high
	Not adequately descriptive of results	Moderate to high

On study design, most studies were descriptive, estimating headache prevalence and/or burden, and had cross-sectional designs adequate for this purpose. An occasionally recurring methodological error seen in some of these was the reporting of “risk factors” despite that cross-sectional studies can provide evidence only of association, not causation [17,18]. A few studies had more analytical aims, explicitly attempting to define causes of or risk factors for headache, and adopted appropriate case-control or cohort designs.

We found studies that had not correctly defined the population of interest for their purposes; instead they surveyed groups of people chosen more for convenience [19-21]. Often these were studies of patient populations. We did not make a register of the very poor studies that attempted to infer population prevalence from patient samples, although there were examples of this.

Not all studies adopted methods of sampling from the population of interest with due concern for bias avoidance. Among studies sampling by telephone, not all clearly recognized, or sought to quantify or manage, the risk of bias arising from uneven distribution of telephones across different age, gender and socioeconomic groups [22-39]. Among those making contact with households, either by telephone or visiting, not all explicitly avoided selection bias arising when the person interviewed was whoever happened to answer. Some studies did not adequately describe their sampling methods – how households or individuals were selected such that a selection probability could be assigned to them.

We found headache surveys with sample sizes clearly too small for their stated purposes. We did not register these. In many cases, sample sizes were greatly reduced by high levels of non-participation [40-45]. We found, more seriously because no remedy could be offered, other studies in which levels of participation were not reported [20,21,33,37,38,46-79].

Access methods in published studies included visits to households and calls to telephones (land-line or mobile), usually in either case without prior warning (cold-calling). The methods were not always adequately described: in particular, accounts were rarely given of the management of apparently empty households or of unanswered telephone calls. Also used were access by mail or e-mail [40]. Participation levels were invariably low in such studies, such that adequate management of selection bias was virtually impossible. Some studies summoned prospective participants to the interviewer [80,81], again not always with adequate management of selection bias.

The great majority of published studies applied case definitions in some manner according to the criteria of ICHD-II [82] or the earlier ICHD-I [83]. Not all did so in the same way: “migraine” in some included all of its subtypes (ICHD-II codes 1.1-1.6) but in others only migraine with or without aura (ICHD-II codes 1.1 and 1.2). The terms “migraine” and “tension-type headache” included both episodic and chronic forms of each, or were restricted to the episodic forms, with the chronic forms subsumed within the category of headache occurring on ≥15 days/month (often referred to as “chronic daily headache”). Several studies separately reported the prevalences of migraine and probable migraine [84-88], establishing case definitions implying these were distinct entities. A few studies did not explicitly follow ICHD-II or ICHD-I [78,89-93]. As to timeframe, most studies reported 1-year prevalences. Some reported lifetime prevalence [26,32,37,44,49,50,54,58],[64,69,70,80,81,94-108] and others reported shorter timeframes [28,31,80,81,90,109-112] (*e.g.*, 3 months). A considerable number reported no timeframe [21,39,41,42,46,51,52,57],[63,66,71,73,75-79,92,93,108],[113-123].

Most studies used a two-stage procedure for case ascertainment: participants were first asked whether they had headache or not (screening question) and only those who did were posed the diagnostic questions. Most studies then applied structured diagnostic questionnaires. Usually these were explicitly “based on ICHD-II” (or ICHD-I), but many studies referred to “modifications” of these criteria without indicating what these were. Not all studies reported diagnostic validation. Some did but attempted validation among headache patients in clinic rather than in a sample drawn from the population of interest [5,89,124-126].

We found few studies conducting burden estimations that were both relevant and comprehensive [3-5,40]. Many studies reported only prevalence; some reported symptom burden (usually in terms of frequency, intensity and duration); some included limited enquiry into functional impact (commonly using the migraine disability assessment (MIDAS) questionnaire [127]). A very few reported burden on others [41,128] and four estimated societal economic impact [9,129-131]. Only three [4,5,40] explicitly addressed the limitations of recall, a factor inherent in enquiries into burden over past periods of time (often three months).

All of these methodological shortcomings could be, and often were, compounded by deficiencies in quality of reporting.

## Discussion

In the following, under the various themes, we discuss the issues of concern and highlight how methodological shortcomings might have detrimental impact on study quality.

### Study design

Most published studies used appropriate designs. This might have been because most were of headache prevalence, requiring simpler cross-sectional designs. There was a tendency in some to overstep the limitations of these designs, which cannot for example produce evidence of causation to identify risk factors. The few studies that explicitly set out to define causal or risk factors for headache recognized the need for case-control or cohort designs.

### The population of interest

In headache research, the population of interest is usually the population of a whole country, because national data are needed for health policy, but there may be good reasons to study regions larger [40] or smaller [5] than a country. Depending on the aim of the study, sub-populations defined by additional characteristics may be perfectly legitimate subjects of study: specific age groups (*e.g.*, adults of working age, adolescents, school or pre-school children); members of groups defined by ethnicity, culture or language; workers in certain trades or professions, or university students; people with another particular disease, *etc*. Headache *patient* populations on the other hand, while they are easily accessed, are rarely of interest: they are highly selected and, furthermore, the criteria by which they are selected (often self-selected) are generally indeterminable. A study of such populations tells little about, and cannot be extrapolated to, either the general population or any more broadly-defined population.

### Sampling, and bias avoidance

It is rarely possible to survey everyone in the population of interest, and usually necessary to choose from that population a smaller, manageable sample of people to whom access is possible. Because the intention is to generalize the data from this sample to the whole population of interest, the essential requirement is that it should remain representative of the population of interest. “Representative” means similar to the population of interest in all properties of relevance to (*i.e.*, likely to influence) the object of measurement (here, headache prevalence and/or burden). In the context of headache, representativeness clearly encompasses age and gender, which are known to affect headache prevalence, and probably should encompass socioeconomic class, employment status, area of habitation (rural or urban), ethnicity and possibly native language and/or tribal group.

Random sampling from the entire population of interest, either simple or stratified, depends upon chance to achieve representativeness, with likelihood of success dependent on sample size. In most cases, random sampling depends on the existence of some form of overview of the population: for example, census data, population registers or a map showing all households in an area to be sampled. Studies adopting this method usually sampled by telephone: an established methodology, offering cheap and easy access. In addition, this method is possible in the absence of a population overview such as a complete telephone directory through the technique of random digit-dialling (dialling area code(s) followed randomly by as many digits as are typical for phone numbers in the area(s)) [132]. Telephone sampling can work well in countries where telephones (land-line or mobile) are both widespread and evenly distributed across different age, gender and socioeconomic groups; otherwise, it is open to serious selection bias.

Many studies employed some form of cluster sampling, usually selecting participants from a limited number of defined geographical areas (*e.g.*, blocks or streets, or parts of villages), themselves chosen randomly. This method is logistically efficient when selected participants are to be visited. In countries with obligatory schooling, representative samples of children of school age can be obtained by selecting all, or a random sample, of the pupils of a representative sample of schools (a form of cluster sampling).

Generally, in headache studies, only one member of a family is selected. Members of families are similar genetically, share their environment and have common lifestyles; these factors effectively reduce variance if two or more are included. Special issues therefore arise when sampling is dependent upon contacts with households rather than single persons (*e.g.*, calling door-to-door or by household telephone without prior warning). Within a household, certain types of person are more likely to stay at home, open the door or answer the phone: selection bias will inevitably arise when the person interviewed is whoever happens to answer.

#### Sample size determination

Sample-size requirement depends on the prevalence of the disorder and the precision of the estimates needed. Table 2 shows the margins of error for a prevalence of 10% (taken as an example) associated with sample sizes ranging from 200 to 10,000. By increasing the sample size from 200 to 2,000, the margin of error is decreased from 4.2% to 1.3% (absolute), but the gain from sample sizes of >2,000 is relatively small.

**Table 2:** Margin of error (95% confidence interval) according to sample size

**Sample size (N)**	200	500	1,000	2,000	5,000	10,000
**Margin of error (%)**	4.2	2.7	1.9	1.3	0.8	0.6

A larger sample may be needed to estimate burden than to estimate prevalence, because burden is not distributed equally among cases: most of it is accounted for by a minority of those with the disorder. In a Swedish survey, for example, 27% of people with migraine reported 68% of all attacks [133]. Among all people with migraine, TTH or MOH, the relatively few with MOH have the highest individual burden [9].

#### Participation and non-participation

Sampling merely selects intended subjects, who become participants only when accessed and engaged (which entails procuring their willing cooperation so that the enquiry can be completed). A high level of non-participation is potentially damaging to representativeness, although how much so depends on the factors responsible for it [134]. Non-participation results mostly from outright refusal: a key factor in headache studies is that people with headache, having a personal interest, are more likely to participate, promoting a form of selection bias that can be highly misleading. Also in headache studies, non-participation is not a constant between important subgroups (*e.g.*, young males are often least willing).

As an access method, visits to households permit face-to-face interviews, which is the most direct method of engagement, allowing physical examination where this is a necessary part of the enquiry. Telephone interviews are almost as direct, without allowing physical examination. Access by mail is cheap, and by e-mail even cheaper, but both methods presume the use of self-administered questionnaires (also cheap, but requiring a high degree of literacy). The lack of engagement with an interviewer provides no encouragement to respond and no opportunity for clarifications; participation rates are generally low, incomplete returns are common and selection bias is unavoidable because certain types of people are inherently less likely to reply. Summoning prospective participants to the interviewer is time-consuming for them and invites bias with regard to who are willing and have the time.

### Caseness and ascertainment

In studies whose purpose is to assess headache prevalence, or describe its characteristics, case definition – what precisely is meant by “headache” (or its types and subtypes, when these are to be considered) – is of obvious and fundamental importance. The criteria of ICHD-II [82] are currently the common language of definition, and description, of headache disorders.

Caseness must have a timeframe. This said, inconsistent terminology in headache has arisen, largely because many headache disorders are chronic but with episodic manifestations. An “active headache disorder” is, by definition, characterized by the occurrence of symptoms within the previous year [82]. Prevalence studies that adopt this definition of a case (*i.e.*, an individual who reports at least one headache episode during that time) necessarily use a timeframe of 1 year and usually report the findings as “1-year prevalence”. Strictly speaking, these are estimates of the number of *current* cases (*point* prevalence). A different enquiry that defines a case only when symptoms are actually present (“headache now” or “headache yesterday” [40]) also estimates *point* prevalence, but of headache attacks or headache as a symptom rather than of a headache disorder. We found most studies reported 1-year prevalence. Other timeframes, especially lifetime prevalence, are relevant to particular purposes (*e.g.*, genetic studies). Shorter timeframes (*e.g.*, 3 months) are difficult to relate, whilst studies specifying no timeframe lack useful case definition.

The two-stage procedure for case ascertainment (only participants responding affirmatively to a screening question are posed the diagnostic questions) is time saving, but with a potential penalty: a negative answer to the screening question terminates further enquiry, even though it can be false.

For epidemiological purposes, diagnostic criteria are almost always built into a structured questionnaire, although this is not how diagnoses are usually made in clinic. ICHD-II criteria were not designed for epidemiological enquiry, and are not particularly well-suited to it. For several reasons, modifications of ICHD-II *are* almost invariably necessary. First, their strict application would require that all participants be personally interviewed and in many cases examined by a competent clinician. In most cases, even if possible, this would be a questionable use of resources. Second, ICHD-II criteria are expressed in technical language, and must be translated for lay participants without loss or distortion of meaning. Third, certain criteria distinguishing between migraine and TTH pose particular problems in epidemiological surveys, noted empirically in studies within the Global Campaign [3-5]. It has been found difficult to gather correct responses on headache duration, requiring patients to consider *untreated* attacks, which they may never have or last had long ago. There are no easy lay explanations of photo- and phonophobia, which are technical concepts, and it is even more difficult to specify what degrees of photo- and phonophobia fulfil migraine criteria in ICHD-II.

The separate reporting of prevalences of migraine and probable migraine (or TTH and probable TTH), as though these were distinct entities, is highly problematic. ICHD-II sets the general rule that, when all but one criterion for disorder X are met, the diagnosis is “probable X” (*provided that* not all criteria are met for another disorder Y, in which case the correct diagnosis is Y). This has an important purpose in clinical management, providing a basis for a treatment plan pending later diagnostic confirmation. In epidemiological surveys, later confirmation is not expected: initial diagnoses of probable X have no opportunity to be amended either to X or to another diagnosis. In the specific cases of migraine and TTH, diagnosis of the former depends upon the presence of specific features (*e.g.*, nausea, vomiting, photophobia and phonophobia, aggravation by physical activity), while diagnosis of TTH depends essentially upon the absence of these same features. By ICHD-II rules, the presence of all but one feature of migraine is not consistent with a diagnosis of TTH, and must lead to a diagnosis of probable migraine. The same is true of probable TTH. Yet there are unavoidable uncertainties in questionnaire-based diagnoses, with the consequence that, according to ICHD rules, about half of such diagnoses are probable while, in validation studies conducted in sub-groups of the same populations, fewer than 10% of expert diagnoses are probable (unreported data from Ayzenberg *et al. *[3], Yu *et al. *[4] and Rao *et al. *[5]). It is difficult to see that the concept of “probable X” (as something distinct from “X”) serves any purpose in studies of population health.

#### *Validation of the diagnostic questionnaire*

Validation proves the diagnostic capability, in the population of interest, of the diagnostic questionnaire. It gains importance when modifications of ICHD-II criteria are adopted, which is almost always necessary (see above). It must, to achieve its purpose, be performed either in a separate sample of the population of interest, selected by identical methodology to the main sample, or in a randomly or consecutively selected sub-sample of participants in the main study. Headache patients in clinic are especially unrepresentative of any population of interest in terms of their headache disorders and, probably, a range of other relevant factors. They commonly have more knowledge of headache, and perform differently from non-clinic populations when answering questionnaires because they have rehearsed their histories.

**Validation is not always possible.** In countries where there are no headache experts, there is no gold standard available. In such cases only, going ahead without diagnostic validation may be justifiable, because the alternative is that research of public-health importance can never be commenced. What should then be avoided is the invention of a new and untested questionnaire, rather than adopt one that has at least been used previously and validated in multiple languages and cultures.

### Burden estimation

This area of enquiry is being developed by LTB in Global Campaign studies in Russia [3], China [4], India [5], Pakistan [6], Saudi Arabia, Zambia, Ethiopia, Nepal and Guatemala (not yet published). “Relevant” implies that measured burden must be attributable to headache, and not to any other cause (including comorbidity). Burden of headache has many different elements. “Comprehensiveness” requires that *all* are measured if a full account is needed. However, the purpose of the study can legitimately restrict the enquiry to specific elements (for example, financial burden [9]).

Some elements of burden are not quantifiable, but amenable only to qualitative (descriptive) analysis. In all cases of burden estimation (more so than for prevalence estimation because of the complexities of burden), the limitations of *recall* are an important factor in the generation of information bias.

#### *Elements of burden*

*Symptom burden* in common headache disorders arises from pain, and, in migraine, additionally from nausea, vomiting and photo- and/or phonophobia. While present, these symptoms may cause debility and prostration, and reduce functional ability. The consequences include inability to work. This secondary *disability burden* is magnified because headache is most common in people between their teens and 50-60 years of age – the productive years. Attached to disability may be a *cost burden* from lost pay. To the extent that this arises from lost wages, as a consequence of absenteeism, it is a relevant and important component of the burden of headache for many people. However, housewives and unemployed people may have no income to lose. In other cases, this cost is only a small part of the personal burden because it is largely borne by employers or insurers, contributing instead to the very high *societal economic burden*.

Headache attacks are unpleasant; people who experience them frequently worry about when the next may occur. They may identify triggers, and attempt to eliminate them by lifestyle compromise. Leisure activities may be cancelled or curtailed because of headache; when many social events have been cancelled, they are likely not to be planned in the first place. These are all elements of *interictal burden*, which may be sufficient to impair quality of life. The great importance of interictal burden lies in the fact that it is continuous, rather than present only during attacks occurring perhaps every 30 days. This means both that it should not be ignored and that, if over-estimated, it will greatly distort overall burden quantification.

A consequence of recurring inability to work may be decreased probability of promotion, with failure to develop full career potential. A consequence of lost school-time may be reduced career opportunities. In both cases, the result is lower pay and impaired financial security. Over a lifetime, the *cumulative burden* of financial losses can be substantial.

A summary measure of *overall individual burden* is unlikely to be comprehensive, but the concept is attractive for its simplicity. Measures of quality of life are very non-specific, but comparison with a matched non-headache group may provide a useful indicator of burden expressed in very broad terms.

*Burden on others*, unaffected by headache themselves, arises in several ways. Employers and work colleagues carry part of it when paid-for work is not done: either the employer pays for nothing, or colleagues take on extra duties to make up. Family and friends lose the companionship they reasonably expect, but which is not given by a person shut away in a dark room. Children may not always be looked after. Partners and other family members may inherit increased shares of chores and responsibilities. They may acquire a carer burden, called to look after the person with headache. Carers, as well as the sufferer, can lose time from work.

Health-care resource consumption, when direct treatment costs for a condition affecting a large proportion of the population are reimbursed by a state-funded health system, is a substantial contributor to *societal economic burden*. But by far the greater part of the financial cost of headache is the indirect cost of absenteeism and reduced effectiveness at work [9,130].

### Reporting of methods and results

Methods can be assessed only according to how they are reported. While we found that methodological shortcomings were often compounded by deficiencies in quality of reporting, it might sometimes be the case (but this can never be known) that methods were better than the reporting of them indicated.

The same is true of results.

## Conclusions

There is worldwide neglect of headache disorders as major causes of public ill-health, and inadequate responses to them in countries throughout the world [135]. In this context, population-based studies of headache inform needs assessment and are the essential guide for provision of headache services and the commitment of appropriate resources to them. Quality assurance in the design, conduct and reporting of them is as important as in clinical trials, but this fact has not been well recognized. The literature reveals many issues, highlighted here, that require attention.

A full account of burden, which should underpin needs assessment, requires a rather detailed enquiry. The methodology is still under development.

The production, publication and dissemination of expert guidance, empirically tested, would be of major benefit – a large step towards improving quality in population surveys of headache prevalence, burden and cost. It is a necessary step in the path towards addressing these inadequate responses.

## Competing interests

TJS, LJS, GLB, RJ, ZK and PM are directors and trustees of *Lifting The Burden*. TD and SC are staff members of the World Health Organization (WHO); they alone are responsible for the views expressed in this publication, which do not necessarily represent the decisions, policy or views of WHO.

## Authors’ contributions

TJS and LJS conceived the idea and assembled the group of experts. LJS and TJS reviewed the literature and LTB-supported population-based studies. All authors were members of the expert consensus group and contributed to the extraction of methodological themes and identification and discussion of methodological shortcomings. TJS and LJS drafted the manuscript. All authors reviewed the manuscript in various drafts and approved the final version.
